# Risk factors for acute chemical releases with public health consequences: Hazardous Substances Emergency Events Surveillance in the U.S., 1996–2001

**DOI:** 10.1186/1476-069X-3-10

**Published:** 2004-10-20

**Authors:** Perri Z Ruckart, Wendy A Wattigney, Wendy E Kaye

**Affiliations:** 1 Agency for Toxic Substances and Disease Registry, Division of Health Studies, Epidemiology and Surveillance Branch, 1600 Clifton Road, MS E-31, Atlanta, Georgia, USA

## Abstract

**Background:**

Releases of hazardous materials can cause substantial morbidity and mortality. To reduce and prevent the public health consequences (victims or evacuations) from uncontrolled or illegally released hazardous substances, a more comprehensive analysis is needed to determine risk factors for hazardous materials incidents.

**Methods:**

Hazardous Substances Emergency Events Surveillance (HSEES) data from 1996 through 2001 were analyzed using bivariate and multiple logistic regression. Fixed-facility and transportation-related events were analyzed separately.

**Results:**

For fixed-facility events, 2,327 (8%) resulted in at least one victim and 2,844 (10%) involved ordered evacuations. For transportation-related events, 759 (8%) resulted in at least one victim, and 405 (4%) caused evacuation orders. Fire and/or explosion were the strongest risk factors for events involving either victims or evacuations. Stratified analysis of fixed-facility events involving victims showed a strong association for acid releases in the agriculture, forestry, and fisheries industry. Chlorine releases in fixed-facility events resulted in victims and evacuations in more industry categories than any other substance.

**Conclusions:**

Outreach efforts should focus on preventing and preparing for fires and explosions, acid releases in the agricultural industry, and chlorine releases in fixed facilities.

## Background

Recent high-profile hazardous materials incidents, such as an explosion at a pharmaceutical supply plant in Kinston, North Carolina [1] and an explosion at a manufacturing plant that makes automotive insulation products in Corbin, Kentucky [2], highlight the need to determine risk factors for hazardous materials incidents to reduce the public health consequences from uncontrolled or illegally released hazardous substances. Previous analyses of risk factors for chemical releases have been restricted to one state, one chemical, or only a few years of data. An analysis of hazardous substance releases in Wisconsin found that ammonia releases occurred more frequently in the food processing, manufacturing, and agricultural industries; and ammonia releases were more likely than releases of all other chemicals to result in evacuation and injury [3]. Burgess et al. showed that chemical releases in Washington state inside buildings and releases involving three to five victims were more likely to require evacuation or sheltering in place [4]. An analysis of hazardous substance releases during 1990–1992 showed that ammonia, chlorine, and acids were frequently released and were more likely than other substances to result in injuries or evacuations [5].

A more comprehensive analysis is needed to determine which risk factors are associated with releases of hazardous substances that result in victims or evacuations. The Hazardous Substances Emergency Events Surveillance (HSEES) system, maintained by the Agency for Toxic Substances and Disease Registry (ATSDR), is an active, multistate surveillance system designed to collect and analyze information about acute releases of hazardous substances. Data elements captured in HSEES include time, date, and day of the week when the event occurred; event type (fixed-facility or transportation-related event); geographic information; factors contributing to the release; type of chemicals released; information about injured persons (victims); industry responsible for the event; and information about decontaminations and orders to evacuate. Data from HSEES were analyzed to identify potential risk factors associated with chemical releases involving victims or evacuations during 1996–2001.

## Methods

### Surveillance

HSEES events are defined as sudden, uncontrolled, or illegal releases of at least one hazardous substance that had to be removed, cleaned up, or neutralized according to federal, state, or local law. A substance is considered hazardous if it might reasonably be expected to cause adverse human health outcomes. Threatened releases are also included in HSEES, if the amount threatened to be released would have required removal, cleanup, or neutralization under federal, state, or local law and the threat led to an action to protect the public health (e.g., rerouting traffic, closing a road, or ordering an evacuation). Events involving only petroleum are excluded from HSEES.

The purpose of HSEES is to reduce morbidity and mortality associated with acute releases of hazardous substances. The goals of the system are to: 1) describe the distribution and characteristics of hazardous substances emergencies, 2) describe morbidity and mortality of employees, emergency responders, and the general public resulting from hazardous substance releases, 3) identify risk factors for morbidity and mortality, and 4) identify strategies that might reduce future morbidity and mortality resulting from the release of hazardous substances.

The pilot phase of the surveillance system was from January 1, 1990 through December 31, 1992. Data for 1996–2001, the most recent period for which complete data are available, were analyzed. Thirteen states participated in HSEES during the entire period analyzed: Alabama, Colorado, Iowa, Minnesota, Missouri, Mississippi, New York, North Carolina, Oregon, Rhode Island, Texas, Washington, and Wisconsin. An additional four states participated during portions of that period: Louisiana (2001), New Hampshire (1996), New Jersey (2000–2001), and Utah (2000–2001).

State health department personnel used a variety of sources (e.g., records and oral reports of state environmental agencies, police and fire departments, and hospitals) to collect information about the hazardous events. Before January 1, 2000, data were entered into a computerized data-entry system designed by ATSDR and were transmitted quarterly to ATSDR for quality control checks and analysis. Beginning on January 1, 2000, data were entered into a web-based application that enabled ATSDR to instantly access the data.

A standardized data-collection instrument was used to obtain information on each event. ATSDR provided the states with a training manual to ensure uniformity. A victim is defined as a person experiencing at least one documented adverse health effect (such as respiratory irritation or chemical burns) that probably resulted from the event and occurred within 24 hours after the release. The HSEES system does not identify the immediate cause of the adverse health effect other than the event itself. Official evacuations were recorded in the system if they were ordered by on-scene coordinators such as a fire or police chief, a member of a HazMat team, or some other type of official. Reasons for the evacuation are not captured.

Industry codes for the type of industry responsible for each HSEES event were assigned according to the 1990 Industrial Classification System of the United States Census Bureau [6]. The industry classification system consists of 243 codes. From this, 18 major industry categories were defined as follows: agriculture, forestry, and fisheries; construction; mining; manufacturing chemical and allied products; manufacturing petroleum and coal products; manufacturing (excluding chemical and allied products and petroleum and coal products); transportation; communications; utilities and sanitary services; wholesale trade; retail trade; finance, insurance and real estate; business and repair services; personal services (comprising industries such as private households, hotels/motels, dry cleaners, and beauty parlors); entertainment and recreation services; professional and related services (which includes hospitals and schools); public administration; and military. Individual chemicals that were released or threatened to be released were assigned to one of 11 substance categories (acids, ammonia, bases, chlorine, other inorganic substances, paints and dyes, pesticides, polychlorinated biphenyls, volatile organic compounds, mixtures of substances from different categories mixed before release, and other). Events that involved chemicals from more than one substance category were categorized as "multiple substances."

### Analysis

Data were analyzed using SAS [7] logistic regression to identify potential risk factors associated with chemical releases involving victims or evacuations during 1996–2001. Events that involved only a threatened release were excluded from analyses that examined risk factors for events with victims. Fixed-facility and transportation-related events were analyzed separately. Potential risk factors included time of day (0000–0559, 0600–1159, 1200–1759, and 1800–2359), day of week (weekday vs. weekend), and season (December-February, March-May, June-August, and September-November) when the event occurred; type of general land use of the surrounding area in which the event occurred (industrial, commercial, residential, agricultural, other); release type; and substance category.

For each chemical reported, up to two entries for type of release could be selected from six choices (spill, air, fire, explosion, threatened release, other). To describe an event, release type was assigned in a hierarchic arrangement (fire and explosion, explosion, fire, air, spill, or threatened) according to all release types reported for an event. When an event had multiple release types, the highest rank was used to assign the release type. For example, an event that had a chemical released as both an air emission and a fire (or one chemical released by air and one chemical released by fire) would be classified as fire. Threatened releases were analyzed only for events with evacuations. The area of evacuation recorded if the evacuation zone was downwind/downstream of the release, a circular area, the building(s) or affected part of the building(s), both a circular area and downwind/downstream, or if no criteria were used.

For fixed-facility events, industry category and factor contributing to the release (improper mixing or filling; equipment failure; human error; system problem; beyond human control, including power failures and bad weather; illegal dumping or deliberate damage; other) were also included. Although up to two factors could have been selected for each event, we analyzed only the first factor selected, because only 13% of events had a second factor selected and specific combinations of factors were too sparse to provide meaningful analysis.

We examined the likelihood of an event resulting in a victim or evacuation for specific substance and industry categories separately. Substance categories were coded as yes/no; the referent group was all other substances. Similarly, industry categories were coded as yes/no; the referent group was all other industries. Because most transportation-related events were coded into the transportation industry (79%), the association between industry type and an event with a victim (or evacuation) was examined only for fixed-facility events. Mode of transportation, categorized as ground (i.e., truck), rail, water, air, and other, was also included in the analysis for transportation-related events.

To further specify risk factors, multivariate and stratified analyses were performed to examine whether certain combinations of variables significant as univariate predictors were associated with a greater likelihood of an event with a victim or evacuation. A multiple logistic regression model was constructed to examine the potential interactions among time of day, weekday, and season. For fixed-facility events, analysis stratified by industry category examined the association between specific substances and an event with a victim or evacuation within industries.

## Results

During 1996–2001, participating state health departments reported 39,766 events to the HSEES system. The analysis of risk factors for events with at least one victim excluded 568 events that involved only a threatened release, and thus focused on 39,198 events (29,974 (76%) fixed-facility events and 9,224 (24%) transportation-related events). At least one victim was reported for 2,327 (8%) of the fixed-facility events with the number of victims per event ranging from 1 to 259 (mean = 4 and median = 1). Similarly, at least one victim was reported for 759 (8%) of the transportation events with the number of victims per event ranging from 1 to 65 (mean = 2 and median = 1).

The analysis of risk factors for evacuations focused on 39,622 events (30,190 (76%) fixed-facility events and 9,432 (24%) transportation-related events) because information about whether an evacuation was ordered was missing for 144 events. Evacuations were ordered for 2,961 (10%) fixed-facility events with the number of persons evacuated per event ranging from 0 (10 events) to 11,000 (mean = 112 and median = 20). For transportation events, 453 (5%) events involved an ordered evacuation; the number of persons evacuated per event ranged from 0 (3 events) to 8,700 (mean = 163 and median = 20). For fixed-facility events, the area of evacuation was primarily the building(s) or affected part of the building(s) (2,222 events, 75%), while for transportation events, the evacuation zone was primarily a circular area (199, 44%).

### Events involving victims

Emergency chemical releases at fixed facilities involving victims were more likely to occur on weekdays than weekends (prevalence odds ratio [POR] = 1.16, 95% CI = 1.03–1.29) (Table 1). Furthermore, events at fixed facilities with victims were most likely to occur during 1200–1759 (POR = 1.90, 95% CI = 1.61–2.25) compared with the hours of 0000–0559. Transportation-related events were less likely to occur during specific hours or on weekdays. Seasonality was not associated with a higher likelihood of chemical releases with victims for either fixed-facility or transportation-related events.

**Table 1:** Univariate analyses for acute chemical releases involving victims, by type of event.

**Variable**	**Fixed Facilities**			**Transportation**		
	
	**No.**	**% with victims**	**POR (95% CI)**	** No.**	**% with victims**	**POR (95% CI)**
Time of day						
0000–0559	4117	4.3	referent	** **1205	9.8	referent
0600–1159	10104	7.4	1.78 (1.51–2.11)	** **3011	9.2	0.94 (0.75–1.18)
1200–1759	9289	7.9	1.90 (1.61–2.25)	** **2539	8.7	0.87 (0.69–1.11)
1800–2359	5069	7.4	1.78 (1.48–2.14)	** **1171	8.8	0.89 (0.67–1.17)
Day of week						
Weekend	5765	7.0	referent	** **1226	7.0	referent
Weekday	24209	8.0	1.16 (1.03–1.29)	** **7998	8.4	1.22 (0.97–1.54)
Season						
Dec.-Feb.	6643	7.3	referent	** **1631	8.3	referent
Mar.-May	7988	7.9	1.08 (0.96–1.23)	** **2812	7.4	0.88 (0.70–1.10)
Jun.-Aug.	8480	8.1	1.11 (0.99–1.26)	** **2794	8.9	1.08 (0.87–1.35)
Sep.-Nov.	6863	7.7	1.07 (0.94–1.21)	** **1987	8.5	1.02 (0.81–1.30)
Area*						
Industrial	18936	5.6	0.59 (0.45–0.77)	** **2547	12.7	0.20 (0.15–0.27)
Commercial	5336	3.4	3.49 (2.67–4.57)	** **3385	2.9	0.46 (0.36–0.59)
Residential	2097	17.3	1.68 (1.53–1.85)	** **612	6.3	1.08 (0.98–1.19)
Agricultural	2253	22.1	1.70 (1.27–2.29)	** **1628	15.5	1.29 (1.02–1.64)
Other	1064	9.2	referent	** **903	15.9	referent
Release Type						
Spill	11165	6.2	referent	** **8020	6.5	referent
Air	17280	7.1	1.16 (1.06–1.28)	** **945	18.3	3.25 (2.69–3.91)
Fire	878	22.8	4.47 (3.75–5.33)	** **134	35.8	8.08 (5.62–11.64)
Explosion	244	53.7	17.57 (13.51–22.86)	** **11	45.5	12.07 (3.67–39.68)
Fire and explosion	98	57.1	20.21 (13.45–30.38)	** **5	40.0	9.66 (1.61–57.91)
Substances^†,‡^						
Acid	1832	14.8	2.20 (1.92–2.53)	NA		
Ammonia	2150	12.6	1.81 (1.58–2.07)	** **280	17.5	2.46 (1.79–3.38)
Chlorine	597	28.0	4.90 (4.07–5.89)	** **18	33.3	5.62 (2.10–15.01)
Other inorganics^§^	NA			** **968	10.7	1.40 (1.23–1.74)
Pesticides	810	14.8	2.13 (1.74–2.59)	NA		
Multiple substances^¶^	1142	24.0	4.12 (3.57–4.75)	** **352	36.4	7.46 (5.92–9.41)

*Type of general land use of the surrounding area in which the event occurred.

^†^Only substance categories with a 95% CI greater than 1.0 are presented.

^‡ ^Substance categories were coded as yes/no. The referent group is all other substances.

§Excludes acids, bases, ammonia, and chlorine.

^¶^Events with more than one hazardous substance released from different chemical categories.

Fixed-facility events with victims were most likely to occur in areas described as commercial, than in the referent group "other," while transportation-related events were more likely to occur in agricultural areas. Compared with spills, fire and explosion was the release type with the greatest likelihood of victims for fixed-facility events (POR = 20.21, 95% CI = 13.45–30.38). Releases of chlorine (POR = 4.90, 95% CI = 4.07–5.89) were most likely to result in fixed-facility events with victims compared with releases from all other chemical categories while releases of multiple substances from different categories (POR = 7.46, 95% CI = 5.92–9.41) were most likely to result in transportation events with victims.

Type of industry was examined as a potential risk factor for victims in fixed-facility events only. Industries classified as personal services were most likely to result in events with victims than were releases not involving these industries (POR = 8.24, 95% CI = 7.20–9.44) (Table 2). Factors identified as illegal dumping or deliberate damage were most likely to contribute to a fixed-facility event with at least one victim (POR = 15.93, 95% CI = 10.41–24.36) compared with "other" factors. Factors classified as "other" included maintenance, vehicular accident, fire, explosion, and factors that could not be classified into an existing category.

**Table 2:** Univariate analyses for acute chemical releases in fixed facilities, by events with victims and evacuations.

**Variable**	**Victims**			**Evacuations**		
	
	**No.**	**% with victims**	**POR (95% CI)**	**No.**	**% with evac.**	**POR (95% CI)**
Industry*,^†^						

Agriculture^‡^	578	17.8	2.75 (2.21–3.42)	NA		
Business and repair services	265	23.0	3.76 (2.81–5.02)	268	15.7	1.74 (1.25–2.42)
Communications	33	21.2	3.33 (1.45–7.68)	NA		
Construction	319	15.4	2.26 (1.67–3.08)	NA		
Entertainment and recreation services	196	29.6	5.29 (3.88–7.21)	200	29.5	3.96 (2.91–5.38)
Finance, insurance & real Estate	108	37.0	** **7.36 (4.97–10.91)	107	41.1	6.59 (4.47–9.70)
Manufacturing^§^	4594	9.6	1.39 (1.24–1.55)	4598	18.5	2.59 (2.38–2.83)
Personal services	1025	36.3	8.24 (7.20–9.44)	1064	23.7	3.03 (2.62–3.51)
Professional and related services	953	25.6	4.64 (3.99–5.41)	978	42.3	7.82 (6.85–8.94)
Public administration	273	22.7	3.70 (2.77–4.92)	277	22.4	2.72 (2.05–3.62)
Retail trade	451	33.0	6.45 (5.27–7.88)	456	36.6	5.65 (4.65–6.86)
Wholesale trade	1061	10.7	1.49 (1.22–1.82)	NA		
Factors						
Beyond human control^¶^	1076	2.2	0.58 (0.37–0.92)	1084	8.9	1.56 (1.19–2.05)
Equipment failure	15193	4.5	1.21 (0.97–1.51)	15186	7.1	1.23 (1.02–1.47)
Human error	5900	14.3	4.28 (3.43–5.35)	5930	15.6	2.97 (2.47–3.57)
Illegal dumping or deliberate damage	1152	26.7	** **15.93 (10.41–24.36)	1295	16.6	2.05 (1.59–2.65)
Improper mixing or filling	771	22.6	7.46 (5.69–9.77)	775	27.0	5.94 (4.71–7.49)
System problem	1838	0.9	0.23 (0.13–0.38)	1833	1.4	0.23 (.015–0.35)
Other	2393	3.8	referent	2425	5.9	referent

*Only industry categories with 95% CI greater than 1.0 are presented.

^†^Industry categories were coded as yes/no. The referent group is all other industries.

^‡^Includes forestry and fisheries.

^§^Excludes chemical and allied products and petroleum and coal products, which were analyzed as separate categories.

^¶^Includes power failures and bad weather.

### Events involving evacuations

Emergency chemical releases at fixed facilities involving evacuations were more likely to occur on weekdays (POR = 1.26, 95% CI = 1.14–1.40) (Table 3). Furthermore, events at fixed facilities with evacuations were most likely to occur during 1800–2359 (POR = 1.69, 95% CI = 1.45–1.97) compared with the hours of 0000–0559. Fixed-facility events that resulted in evacuation orders were slightly more likely to occur during June through August than during December through February (POR = 1.12, 95% CI = 1.01–1.25). Transportation-related events were not more likely to occur during specific hours, on weekdays, or during a particular season.

**Table 3:** Univariate analyses for acute chemical releases involving evacuations, by type of event.

**Variable**	**Fixed Facilities**			**Transportation**		
	
	** No.**	**% with evac.**	**POR (95% CI)**	** No.**	**% with evac.**	**POR (95% CI)**
Time of day						
0000–0559	4130	6.5	referent	1237	4.9	referent
0600–1159	10188	10.4	1.67 (1.45–1.92)	3085	5.1	1.04 (0.77–1.41)
1200–1759	9363	10.2	1.64 (1.42–1.88)	2591	5.4	1.11 (0.82–1.51)
1800–2359	5100	10.5	1.69 (1.45–1.97)	1210	6.1	1.26 (0.89–1.78)
Day of week						
Weekend	5790	5.0	referent	1262	7.7	referent
Weekday	24400	4.8	1.26 (1.14–1.40)	8170	8.8	0.95 (0.73–1.25)
Season						
Dec.-Feb.	6683	9.2	referent	1682	5.4	referent
Mar.-May	8058	9.6	1.04 (0.93–1.17)	2860	4.6	0.86 (0.65–1.13)
Jun.-Aug.	8559	10.3	1.12 (1.01–1.25)	2849	4.7	0.87 (0.66–1.14)
Sep.-Nov	6890	10.1	1.10 (0.98–1.23)	2041	4.8	0.89 (0.67–1.20)
Area*						
Industrial	18958	8.5	0.60 (0.48–0.75)	2579	5.7	0.61 (0.43–0.85)
Commercial	5438	5.2	3.11 (2.49–3.88)	3436	3.5	0.87 (0.63–1.19)
Residential	2158	22.3	1.50 (1.38–1.62)	637	5.0	1.12 (0.98–1.28)
Agricultural	2269	23.6	0.68 (0.51–0.89)	1681	7.9	0.86 (0.60–1.22)
Other	1089	5.9	referent	947	4.9	referent
Release Type						
Spill	11129	6.9	referent	7999	3.1	referent
Air	17222	9.3	1.38 (1.27–1.51)	945	12.7	4.55 (3.61–5.72)
Fire	869	35.7	7.48 (6.40–8.75)	134	20.2	7.89 (5.08–12.25)
Explosion	242	36.8	7.85 (5.98–10.29)	10	20.0	7.81 (1.65–36.99)
Fire & explosion	96	46.9	11.90 (7.92–17.89)	4	25.0	10.42 (1.08–100.5)
Threatened	335	34.9	7.24 (5.72–9.17)	231	20.8	8.20 (5.82–11.54)
Substances^†,‡^						
Acid	1850	9.6	1.46 (1.27–1.67)	** **NA		
Ammonia	2144	24.6	3.44 (3.09–3.83)	290	18.3	4.89 (3.57–6.69)
Chlorine	599	33.1	4.79 (4.03–5.71)	22	27.3	7.52 (2.93–19.31)
Multiple substances^§^	1213	25.6	3.41 (2.98–3.90)	386	14.8	3.79 (2.81–5.10)

*Type of general land use of the surrounding area in which the event occurred.

^†^Only substance categories with a 95% CI greater than 1.0 are presented.

^‡ ^Substance categories were coded as yes/no. The referent group is all other substances.

^§^Events with more than one hazardous substance released from different chemical categories.

Fixed-facility events with evacuations were most likely to occur in areas described as commercial compared with the referent group "other." Area type was not associated with ordered evacuations for transportation-related events. The release type fire and explosion was most likely to result in fixed-facility events with evacuations compared with spills (POR = 11.90, 95% CI = 7.92–17.89). Releases of chlorine were most likely to result in events with evacuations compared with releases from all other chemical categories for both fixed-facility and transportation-related events (POR = 4.79, 95% CI = 4.03–5.71 and POR = 7.52, 95% CI = 2.93–19.31, respectively).

We examined type of industry as a potential risk factor for evacuations in fixed-facility events only. Industries classified as professional services were most likely to result in events with evacuations compared with releases not involving these industries (POR = 7.82, 95% CI = 6.85–8.94) (Table 2). Factors identified as improper mixing or filling were most likely to contribute to releases with evacuations in fixed facilities (POR = 5.94, 95% CI = 4.71–7.49) compared with "other" factors.

### Multivariate and stratified analyses of fixed-facility events

Results from the multiple regression analysis of temporal variables indicated an association of borderline significance between fixed-facility events with victims and the three-way interaction indicated by time of day (0600–1159), day of week (weekday), and season (March through May) (p = 0.06); no interaction among temporal variables was indicated for evacuations.

The likelihood of an event with at least one victim or evacuation in fixed facilities was examined for substances within industry category (Table 4). The strongest association was observed for acid releases in the agriculture, forestry, and fisheries industry for fixed-facility events involving victims (POR = 7.28, 95% CI = 2.02, 26.30). Releases of acids, chlorine, and multiple substances in the manufacturing industry had an elevated likelihood of both an event with at least one victim and an evacuation. There were fewer associations between substances and industry categories for events with evacuations compared with events with at least one victim.

**Table 4:** Multivariate analyses* for acute chemical releases in fixed facilities, by industry and substance category.

**Industry**	**Acids**	**Ammonia**	**Chlorine**	**Pesticides**	**Multiple Substances**
	
	**POR (95% CI)**	**POR (95% CI)**	**POR (95% CI)**	**POR (95% CI)**	**POR (95% CI)**
**Victims**					

Agriculture^†^	7.28 (2.02–26.30)	2.65 (1.68–4.17)			
Construction				5.92 (1.43–24.50)	
Entertainment & recreation services			5.78 (2.80–11.94)		
Manufacturing^‡^	1.71 (1.27–2.29)		4.03 (2.69–6.05)		2.74 (1.90–3.96)
Personal services					3.21 (2.30–4.47)
Professional and related services	2.43 (1.49–3.99)		2.99 (1.23–7.27)		
Public administration			3.99 (1.47–10.85)		
Retail trade			3.37 (1.08–10.49)		
Wholesale trade	4.19 (2.32–7.59)	2.35 (1.57–3.54)			
**Evacuations**					

Business & repair services					3.84 (1.55–9.48)
Entertainment & recreation services			3.60 (1.77–7.31)		
Manufacturing^‡^	4.31 (3.64–5.10)		3.32 (2.23–4.67)		2.64 (1.94–3.60)
Personal services					3.07 (1.72–5.45)
Retail trade			4.06 (1.23–13.38)		

*Analysis stratified by industry category examined associations between specific substances and events with victims or evacuations within industries for categories that were significant in the univariate analyses.

^†^Includes forestry and fisheries.

^‡^Excludes chemical and allied products and petroleum and coal products, which were analyzed as separate categories.

## Discussion

Almost 40,000 chemical release events were reported to HSEES during the 6-year period included in this analysis. Approximately 8% of the events resulted in almost 12,000 victims, and approximately 9% involved evacuation of more than 325,000 people. Because the public health consequences of chemical releases can be serious and affect large numbers of people, identifying the risk factors more likely to be associated with hazardous substance releases that result in victims or evacuations is important to target appropriate prevention activities.

For fixed-facility events, fire and explosion was the strongest single risk factor for events involving either victims or evacuations. Illegal dumping or deliberate damage also was strongly associated with fixed-facility events resulting in victims. For transportation-related events, explosion was the strongest single risk factor for events involving victims, and fire and explosion was the strongest for events involving evacuations. The observed associations between time of day (0600–2359) and weekday occurrence are likely to be influenced by production intensity, however information on production intensity is not available in HSEES to examine this relationship further. When temporal variables were modeled together, fixed-facility events with victims were more likely to occur on weekday mornings in the spring, which may coincide with the planting season. These results are consistent with a previous analysis that showed that acute hazardous substance releases with victims were more likely during the planting season in Midwestern states [8].

In the stratified analysis of substances within industry category, the strongest association was for acid releases in the agriculture, forestry, and fisheries industry for fixed-facility events involving victims. Previous analyses have shown that acute chemical releases are common in the agricultural industry, nitric acid and sulfuric acid are frequently released in agriculture events, and releases of acids result in significantly higher proportions of releases involving victims [5,8,9].

Chlorine releases in fixed facilities resulted in victims and evacuations in more industry categories than any other substance. This is consistent with an analysis that found chlorine releases had the greatest significant risk of having events with victims and were almost five times more likely than nonchlorine events to involve evacuations [10]. This is not surprising given that chlorine is a strong corrosive agent. Acute health effects of exposure to low levels of chlorine include sore throat, coughing, and eye and skin irritation; exposure to higher levels may cause severe burning of the eyes and skin, rapid breathing, narrowing of the bronchi, wheezing, bluish discoloration of the skin, and lung collapse [11]. Additionally, chlorine is one of the most frequently produced chemical substances in the United States, with more than 30 million tons produced annually [12]. Chlorine is widely used in a variety of processes, such as synthesizing other chemicals and making bleaches and disinfectants.

The HSEES system collected data in only 17 states during 1996–2001. Each state has different reporting requirements for the amount of hazardous substances released that has to be removed, cleaned up, or neutralized; and reporting of events to participating state health departments is not mandatory. Therefore, the total number of events, events with victims, and events with evacuations may be underestimated. However, HSEES is the only federal hazardous substances release database designed specifically to assess and record the public health consequences of hazardous substances emergency events.

The HSEES system captures more events than other federal reporting systems, such as the United States Environmental Protection Agency's (EPA) Risk Management Program (RMP) [13]. EPA's RMP requires all companies that use certain flammable and toxic substances to develop procedures for hazard assessment, prevention activities, and emergency response [14]. Because company-specific information (e.g., name and street address) is not available to HSEES at the federal level, RMP accident history data provides important insight regarding the hazardousness and regulatory practices associated with accidental releases of chemicals at United States manufacturing facilities [15].

These results indicate that prevention activities should focus on ways to reduce fire and/or explosion hazards in facilities that store, use, or manufacture hazardous substances such as distributing material safety data sheets to employees, keeping work areas dust free, and storing chemicals away from ignition sources. Previous analyses of HSEES data have shown that human error and equipment failure are the most frequent causes of acute chemical releases [10,16-18]; releases due to these causes may result in fires and explosions. Facilities should also have chemical inventories and risk management plans available for responders to enhance their response capabilities [19]. Additionally, security measures (e.g., surveillance cameras, chain-link fences, patrol guards, alarms) should be put in place to reduce deliberate chemical releases, such as theft [20]. Prevention activities also should target agricultural industries that use or store acids and industries that store, use, or manufacture chlorine.

Several state health departments participating in HSEES already have developed strategies aimed at reducing releases and injuries associated with chlorine. These activities include distribution of fact sheets to county emergency management agencies, fire departments, other first responders, and industries; and presentations to municipal water directors, engineers, and hotel/motel swimming pool owners and operators.

The predictors of public health consequences associated with acute chemical releases presented in this paper are broad categories of characteristics available in the national data. Future analyses of HSEES data may explore industry-specific root causes of events and risk of events with victims or evacuations for specific industries.

## Conclusion

The results of this analysis should help guide prevention activities aimed at reducing emergency chemical releases and their associated victims and evacuations. Attention should focus on preventing fires and explosions, releases from illegal dumping and deliberate damage, and acid releases in the agricultural industry. Efforts should continue to educate industry, first responders and other users about the potential hazards of chlorine.

## List of Abbreviations

ATSDR, Agency for Toxic Substances and Disease Registry

CI, confidence interval

EPA, Environmental Protection Agency

HSEES, Hazardous Substances Emergency Events Surveillance

POR, prevalence odds ratio

RMP, Risk Management Program

## Competing Interests

The author(s) declare that they have no competing interests.

## Authors' contributions

PZR led the writing of the manuscript and assisted with the statistical analysis. WAW led the statistical analysis and assisted with the writing of the manuscript. WEK conceived of the manuscript and participated in its writing and interpretation. All authors read and approved the final manuscript.

## Pre-publication history

The pre-publication history for this paper can be accessed here:


